# Signal Transduction Protein Array Analysis Links LRRK2 to Ste20 Kinases and PKC Zeta That Modulate Neuronal Plasticity

**DOI:** 10.1371/journal.pone.0013191

**Published:** 2010-10-07

**Authors:** Susanne Zach, Sandra Felk, Frank Gillardon

**Affiliations:** Boehringer Ingelheim Pharma GmbH & Co KG, CNS Research, Biberach an der Riss, Germany; University of Tuebingen, Germany

## Abstract

**Background:**

Dominant mutations in leucine-rich repeat kinase 2 (LRRK2) are the most common genetic cause of Parkinson's disease, however, the underlying pathogenic mechanisms are poorly understood. Several *in vitro* studies have shown that the most frequent mutation, LRRK2(G2019S), increases kinase activity and impairs neuronal survival. LRRK2 has been linked to the mitogen-activated protein kinase kinase kinase family and the receptor-interacting protein kinases based on sequence similarity within the kinase domain and *in vitro* substrate phosphorylation.

**Methodology/Principal Findings:**

We used an unbiased proteomic approach to identify the kinase signaling pathways wherein LRRK2 may be active. By incubation of protein microarrays containing 260 signal transduction proteins we detected four arrayed Ste20 serine/threonine kinase family members (TAOK3, STK3, STK24, STK25) as novel LRRK2 substrates and LRRK2 interacting proteins, respectively. Moreover, we found that protein kinase C (PKC) zeta binds and phosphorylates LRRK2 both *in vitro* and *in vivo*.

**Conclusions/Significance:**

Ste20 kinases and PKC zeta contribute to neuronal Tau phosphorylation, neurite outgrowth and synaptic plasticity under physiological conditions. Our data suggest that these kinases may also be involved in synaptic dysfunction and neurite fragmentation in transgenic mice and in human PD patients carrying toxic gain-of-function LRRK2 mutations.

## Introduction

Parkinson's disease (PD) is the second most prevalent neurodegenerative disorder and is pathologically characterized by the selective loss of dopaminergic neurons in the substantia nigra causing motor dysfunction. Although the etiology of PD is incompletely understood, genetic studies have identified mutations in several genes that segregate with rare familial forms of the disease [1]. Mutations in the PARK8 gene encoding leucine-rich repeat kinase 2 (LRRK2) are the most prevalent cause of autosomal dominantly inherited PD and are characterised by typical brainstem Lewy body pathology [2]. The most frequent mutation, LRRK2(G2019S), is found in the kinase domain and is responsible for approximately 1% of sporadic PD and 5% of familial cases in Caucasians. LRRK2 is a 286 kDa protein containing an N-terminal leucine-rich repeat, a Ras of complex protein (Roc) GTPase domain, a C-terminal of Roc (Cor) region, a kinase domain, and a WD40 protein interaction domain. Several studies have shown that the G2019S mutation enhances kinase activity *in vitro* and that kinase activity mediates degeneration in transfected neurons. Although the kinase domain of LRRK2 exhibits some sequence homology to the mitogen-activated protein (MAP) kinase kinase kinase family, LRRK2 does not seem to act within classical MAP kinase signaling cascades and its upstream regulators / downstream effectors remain to be identified [3], [4]. By protein-protein interaction analysis binding of LRRK2 to the Hsp90/Cdc37 chaperone complex, C-terminus of Hsp70-interacting protein, and parkin has been detected which may influence the folding and degradation of LRRK2 [5], [6]. Potential LRRK2 protein substrates include moesin, eukaryotic initiation factor 4E-binding protein (4E-BP), and tubulin-beta, however their physiological relevance remains unclear [7]. In the present study, we used protein microarrays containing about 260 human signal transduction proteins in order to identify the signaling networks wherein LRRK2 may be active.

## Methods

### Expression and purification of recombinant proteins

Full-length human LRRK2 expression constructs were generated as described in detail elsewhere [8]. The PD-linked LRRK2(G2019S) mutation was introduced by site-directed mutagenesis. LRRK2 constructs were tagged with a StrepII/Flag tag at their C-terminus and cloned into a pFastBac vector for expression in insect cell cultures. Recombinant LRRK2 Bacmid DNA was generated by transformation of competent DH10Bac *E. coli* cells using the Bac-to-Bac expression system according to the manufacturer's protocol (Invitrogen, Carlsbad, USA). Recombinant baculovirus was isolated from Sf9 cell culture supernatant five days after transfection with LRRK2 Bacmid DNA. Viral titer was determined using the BaculoTiter assay kit (Invitrogen, Carlsbad, USA).

Suspension cultures of High Five insect cells were infected with baculovirus at a multiplicity of infection of 10. Two days later cells were lysed in a buffer containing 100 mM Tris, pH 7.4, 130 mM NaCl, 1 mM sodium fluoride, 1% Triton X-100 and protease inhibitor cocktail (Sigma, Taufkirchen, Germany). Recombinant LRRK2 was purified from the supernatant by StrepTactin Superflow affinity chromatography according to the manufacturer's instructions (IBA GmbH, Göttingen, Germany). Purified protein was stored at −80°C in elution buffer (100 mM Tris, pH 8.0, 150 mM NaCl, 1 mM EDTA) containing 10% glycerol, 0.1 mM EGTA, 0.3% Brij-35.

N-terminal GST-tagged human LRRK2 fragments (amino acids 970-2527) were purchased from Invitrogen (Invitrogen, Carlsbad, USA). N-terminal His-tagged MARKK(K57A) was acquired from the Max-Planck-Institute for Structural Molecular Biology (Hamburg, Germany). The kinase-dead MARKK mutant was generated as described elsewhere [9]. Storage buffer was exchanged to kinase assay buffer using Vivaspin 500 columns (Sartorius, Goettingen, Germany). N-terminal His-tagged human PKC zeta was provided by the University of Dundee (UK).

### Kinase substrate identification using protein arrays

Panorama Human Protein Function Array – Signal Transduction (Sigma, Saint Louis, USA) were used according to the manufacturer's protocol with modifications. These arrays contain a set of 259 full-length human signaling proteins tagged with a biotin-carboxyl carrier protein. The tag is biotinylated only when the fusion protein is correctly folded during expression in insect cells and enables oriented immobilization on streptavidin-coated glass slides. Arrays were washed twice for 5 min in 5 ml assay buffer (250 mM Tris-HCl, pH 7.5, 10 mM MgCl_2_, 0.1 mM EGTA, 2 mM DTT, 1 mM NaO_3_V, 0.1% BSA, 20% glycerol and 0.1% Triton X-100)., To prevent interference with the detection of LRRK2-specific signals, autophosphorylation of active kinases spotted on the arrays was completed by pre-incubation in assay buffer containing 100 µM cold ATP only. It should be noted that this pre-incubation step will also preclude detection of a potential LRRK2-mediated phosphorylation of kinase autophosphorylation sites. GST-tagged LRRK2(G2019S) (970-2527) or inactive LRRK2-(D1994A) (970-2527) (Invitrogen, Carlsbad, USA) were diluted in assay buffer containing cold ATP (final concentration 10 µM) and [γ-^33^P]ATP (30 µCi final activity) in a total volume of 120 µl. Kinases (130 nM) or buffer only (negative control) were overlaid on the arrays, covered with HybriSlip™ cover slips (Aldrich, St. Louis, USA), placed in quadriPerm culture vessels, and incubated for 1 hour at 30°C. Subsequently, arrays were washed in 0.5% sodium dodecyl sulfate (SDS) in assay buffer, followed by assay buffer and ultra-pure water. After centrifugation at 800×g for 4 minutes, arrays were exposed to phosphoscreens (GE Healthcare, Munich, Germany) or X-ray films (Kodak, Stuttgart, Germany). The phosphoscreens were scanned on a Typhoon 9400 laser scanner (GE Healthcare, Munich, Germany) at a resolution of 25 µm and analyzed using Genepix Pro 6.0 software (Molecular Devices Corporation, Sunnyvale, USA) followed by ProtoArray Prospector Analyzer 3.0 software (Invitrogen, Carlsbad, USA). Potential kinase substrates are defined as spotted proteins exhibiting a normalized phosphorylation signal greater 150% of the phosphorylation signal observed on the negative control array and greater 2 standard deviations than the median signal/background value for all negative control spots on the array. Further information about protein array content and data analysis are available from http://www.sigmaaldrich.com/etc/medialib/docs/Sigma/General_Information/hpfm4techbulletin.Par.0001.File.tmp/hpfm4techbulletin.pdf.

### Protein-protein binding studies using protein arrays

For protein-protein binding studies, GST-tagged LRRK2(G2019S) (970-2527) was autophosphorylated in assay buffer containing 10 µM cold ATP for 1 h at 30°C. Arrays were washed and equilibrated in assay buffer as described above. Thereafter, arrays were overlaid with autophosphorylated LRRK2 for 1 h at 30°C. Incubation with recombinant GST (GenWay, San Diego, USA) expressed in *E. coli* was tested as control. After washing in 0.5% SDS containing buffer, arrays were blocked 5 min in blocking buffer (phosphate-buffered saline, 0.1% Tween, 20% glycerol, 2% BSA and 1 mM dithiothreitol) twice. Arrays were then incubated with an anti-GST Alexa 488-conjugated antibody (Millipore, Schwalbach, Germany) (1∶250) over night at 4°C. After two washing steps in blocking buffer and one in PBS, arrays were scanned using a Typhoon 9400 laser at 488 nm excitation and 520 nm emission at a resolution of 100 µm. Cy3-labeled reference protein spots which alleviate grid orientation in analysis were scanned at 532 nm excitation and 580 nm emission. Spotted proteins are considered as potential interactors, if the normalized fluorescence signal is greater 150% of the fluorescence signal observed in the negative control assay and greater 2 standard deviations than the median signal/background value for all negative control spots on the array.

### Kinase assay in solution

Kinase assay were performed in 15 µl assay buffer (25 mM Tris-HCl, pH 7.5, 10 mM MgCl_2_, 1 mM EGTA, 2 mM dithiothreitol, 1 mM Na_3_VO_4_) containing 0.25 µg GST-tagged LRRK2, 30 µC_i_ [γ-^33^P]ATP and 100 µM ATP at 30°C for 45 min. Both recombinant His-tagged PKC zeta and His-tagged MARKK(K57A) were tested as substrates (1 µg each). Full-length LRRK2, recombinant GST (GenWay, San Diego, USA), and GST-tagged moesin (University of Dundee, Dundee, UK) were assayed as well. The kinase reaction was stopped with 4× Laemmli buffer, and the samples were heated for 10 minutes to 75°C. Subsequently, proteins were separated on 4–12% sodium dodecyl sulphate, Bis-Tris polyacrylamide minigels (Invitrogen, Karlsruhe, Germany). The gels were dried for 1 h at 80°C in a vacuum gel dryer (Model 583, Biorad, Hercules, USA), and were exposed to phosphoscreens for 24 h at −80°C. Phosphoscreens were imaged on a Typhoon 9400 laser scanner (GE Healthcare, Freiburg, Germany) and quantified using Quantity One software (Biorad, Munich, Germany).

### Immunoprecipitation

Animal work was approved by the Regierungspraesidium Tuebingen (ID 10-004). C75BL/6 mice were killed by cervical dislocation followed by decapitation. The brains were rapidly removed and placed into ice-cold PBS. The brains were homogenized in 1∶5 (w/v) ice-cold lysis buffer (50 mM Tris pH 7.5, 0.27 M sucrose, 1 mM EDTA, 1 mM EGTA, 1 mM Na_3_VO_4_, 5 mM sodium pyrophosphate, 50 mM sodium fluorid, 1% Triton X-100, 0.1% 2-mercaptoethanol, 1 mM benzamidine, 1 mM PMSF) in a Teflon-glass douncer. Lysates were centrifuged at 16000×g for 15 min at 4°C. Aliquots of the supernatants were taken for protein determination using the Bio-Rad protein assay (Biorad, Munich, Germany).

Three mg total protein were used per reaction. Lysate volume was made up to 700 µl with lysate buffer. PKC zeta was immunoprecipitated using an affinity-purified goat polyclonal antibody (Santa Cruz Biotechnology, Heidelberg, Germany). Pre-immune serum was used as negative control. Protein G-agarose beads (Protein G Immunoprecipitation Kit, Sigma, Saint-Louis, USA) were incubated with antibody or pre-immunue serum for 4 h at 4°C under constant agitation. The beads were pelleted by centrifugation at 4000×g for 3 min at 4°C. The supernatants were removed, and the beads were resuspended in 1 ml of lysis buffer. The wash step was repeated once. Thereafter, brain lysates were added and incubated over night at 4°C under constant agitation. Beads were pelleted and washed sequentially with lysis buffer plus 0.5 M NaCl, lysis buffer alone, and PBS. After a final centrifugation step beads were resuspended in 20 µl Laemmli buffer (1% sodium dodecyl sulfate, 100 mM dithiothreitol, 50 mM Tris, pH 7.5) and heated to 50°C for 15 min. The supernantants were separated from the beads by centrifugation in spin columns (Protein G Immunoprecipitation Kit, Sigma, Saint-Louis, USA), heated up to 75°C for 10 min and subjected to gel electrophoresis followed by immunoblotting.

### Gel Electrophoresis and Immunoblotting

Samples were resolved by electrophoresis on 4–12% NuPAGE Bis-Tris gradient gels according to manufacturer's instructions using NuPAGE MOPS running buffer (Invitrogen, Carlsbad, USA). After transfer to nitrocellulose membranes (Protran, Schleicher and Schuell, Dassel, Germany) membranes were blocked for 1 h at 20°C in 5% skimmed milk powder in Tris-buffered saline and 0.1% Tween. Membranes were then incubated overnight at 4°C with either an goat polyclonal antibody against PKC zeta (Santa Cruz Biotechnology, Heidelberg, Germany) or a rat monoclonal antibody against LRRK2 (clone 1E11, GSF, Munich, Germany) (1 µg/ml each). Horseradish peroxidase-conjugated secondary antibodies and enhanced chemiluminescence reagents (ECL kit, GE Healthcare, Freiburg, Germany) were used for detection. Membranes were checked for protein load and protein transfer using a protein staining kit (MemCode, Pierce, Rockford, USA) that reversibly stains for total protein. Densitometric analysis of immunoblots was performed using Quantity One software (Biorad, Munich, Germany).

### Statistical analysis

All experiments were performed at least twice. Statistical significance was determined by performing a two-tailed Student's t-test using GraphPad Prism Version 5.00 (GraphPad software, La Jolla, USA). A p-value<0.05 was considered significant.

## Results

**Figure 1:**
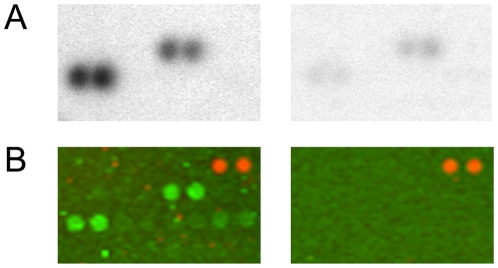
Recombinant LRRK2 binds and phosphorylates arrayed serine/threonine kinase 25. (A) Magnified sections of Panorama Human Protein Function Arrays incubated with GST-tagged LRRK2(G2019S) (amino acids 970-2527) and [γ-^33^P]ATP (left panel) or buffer and [γ-^33^P]ATP (right panel). The phosphoscreen image shows an increase in radioactive signal intensity on the LRRK2-treated protein arrays in the quadruplicate spots of serine/threonine kinase 25. (B) Arrays incubated with GST-tagged LRRK2(G2019S) and cold ATP (left panel) or recombinant GST and cold ATP (right panel). GST-tagged LRRK2 bound to spotted serine/threonine kinase 25 is detected by an Alexa 488-conjugated anti-GST antibody. The GST-treated control array is negative. Orange fluorescence of Cy3-labeled duplicate protein spots is visible in the upper left corner which serve as markers for grid alignment during image analysis.

**Figure 2:**
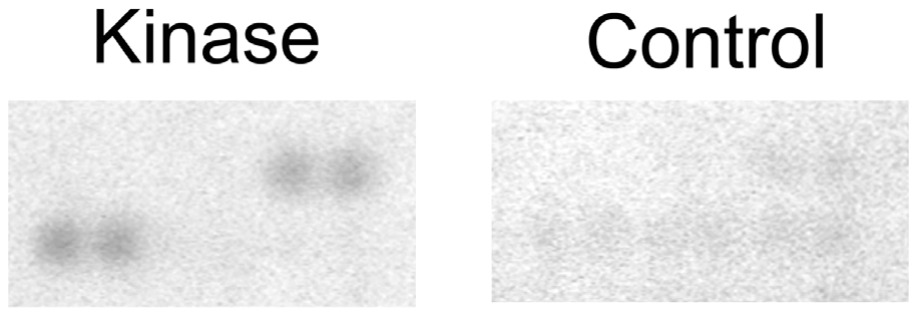
LRRK2 phosphorylates receptor interacting kinase 2 on protein microarrays. Magnified sections of Panorama Human Protein Function Arrays incubated with LRRK2(G2019S) and [γ-^33^P]ATP (left panel) or buffer and [γ-^33^P]ATP (right panel). The quadruplicate spots of receptor interacting kinase 2 exhibit an increased radioactive signal when incubated with recombinant LRRK2(G2019S).

**Figure 3:**
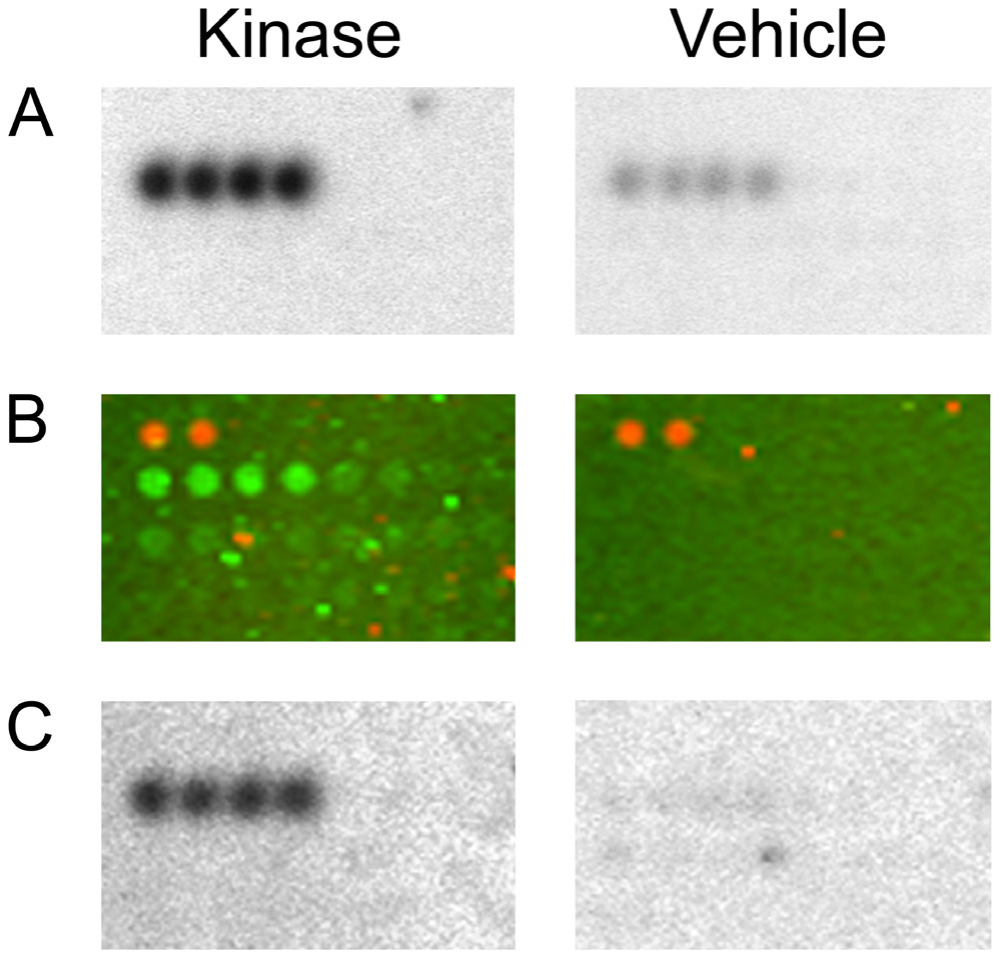
LRRK2 binds to spotted protein kinase C zeta and becomes phosphorylated. Panorama Human Protein Function Arrays incubated with (A) GST-tagged LRRK2(G2019S) and [γ-^33^P]ATP (left panel) or buffer and [γ-^33^P]ATP (right panel), (B) GST-tagged LRRK2(G2019S) and cold ATP or recombinant GST and cold ATP, (C) kinase-inactive GST-tagged LRRK2(D1994A) and [γ-^33^P]ATP or buffer and [γ-^33^P]ATP. (A and C) An increase in radioactive signal intensity is visibly on the LRRK2-treated protein arrays in the quadruplicate spots of protein kinase C zeta. (B) GST-tagged LRRK2 bound to spotted protein kinase C zeta is visualized by an Alexa 488-conjugated anti-GST antibody. Control arrays show background signal only. Orange fluorescence of Cy3-labeled duplicate protein spots is visible in the upper left corner which serve as markers for grid alignment during image analysis.

Panorama Human Protein Functional Arrays were incubated in assay buffer containing [γ-^33^P]-ATP and either GST-tagged LRRK2(G2019S) or buffer only. After several washing steps the arrays were imaged using phosphoscreens and a Typhoon 9400 laser scanner. Genepix Pro digital image analysis followed by ProtoArray Prospector Analyzer statistical analysis (see Table S1) revealed a significant increase in radioactive signal intensity on the LRRK2-treated protein microarrays in the spotted proteins that are listed in Table 1. Four members of the Ste20 serine/threonine kinase family (TAOK3, STK3, STK24, STK25) [10] were identified as potential LRRK2 substrates. LRRK2 has been classified as receptor-interacting protein kinase 7 (RIP7) and *trans*-phosphorylation between RIP kinases has been demonstrated by others [11]. Consistently, receptor-interacting protein kinase 2 (RIP2), the only RIP kinase family member spotted, is phosphorylated by LRRK2. Magnified sections of the arrays are shown in Figure 1 and 2. In addition, increased signal intensity of the quadruplicate spots of PKC zeta on the LRRK2-treated arrays is shown in Figure 3A.

**Table 1:** Proteins spotted on functional human protein arrays showing increased signal intensity following incubation with recombinant LRRK2(G2019S).

Protein	Accession number	Signal/Control
**Protein phosphorylation**		
Thousand-and-one amino acids kinase 3 (TAOK3)	Q9UHG7	2.2
Receptor-interacting serine-threonine kinase 2 (RIPK2)	O43353	2.0
Protein kinase C, zeta (PRKCZ)	Q05513	1.5
Serine/threonine kinase 3 (STE20 homolog, yeast (STK3)	Q13188	1.5
Serine/threonine kinase 24 (STE20 homolog, yeast (STK24)	Q9Y6E0	1.5
Serine/threonine kinase 25 (STE20 homolog, yeast (STK25)	O00506	1.5
**Protein-protein interaction**		
Serine/threonine kinase 25 (STE20 homolog, yeast) (STK25)	Q00506	2.3
Protein kinase C, zeta (PRKCZ)	Q05513	2.1
Heat shock 90kDa protein, alpha (HSPCA)	P07900	1.6
Serine/threonine kinase 24 (STE20 homolog, yeast) (STK24)	Q9Y6E0	1.6
TNF receptor-associated factor 2, transcript variant 1 (TRAF2)	Q12933	1.5

In order to confirm the data from protein array analysis, we first tested whether LRRK2 was able to phosphorylate PKC zeta in a kinase assay in solution, since PKC zeta plays a central role in neuronal physiology [12]. Surprisingly, incubation of recombinant full-length PKC zeta with LRRK2(G2019S) did not result in LRRK2-mediated PKC zeta phosphorylation (data not shown). These findings led us to assume that the radioactive signal on the protein arrays resulted from binding of ^33^P-autophosphorylated LRRK2(G2019S) to spotted PKC zeta. For protein-protein interaction analysis, Panorama Human Protein Functional Arrays were incubated with GST-tagged LRRK2(G2019S) or recombinant GST as control in the presence of cold ATP. Binding of LRRK2(G2019S) to arrayed proteins was analyzed by a subsequent incubation with an Alexa 488-conjugated anti-GST antibody. The arrays were imaged directly on a Typhoon 9400 laser scanner and analysed by Genepix Pro followed by ProtoArray Prospector software (see Table S2). As can be seen from Figure 3B, a significantly higher green fluorescence signal of the quadruplicate PKC zeta spots was detected when compared to the corresponding spots on the control arrays. In addition, STK24 and STK25, which were already identified as potential LRRK2 substrates (shown above), were detected as LRRK2 binding proteins (Table 1; Figure 1B). Interacting proteins also included heat shock protein 90, which has already been identified as LRRK2 binding/stablising protein by others [13], and tumor necrosis factor receptor-associated factor 2 (TRAF2) (Table 1).

**Figure 4:**
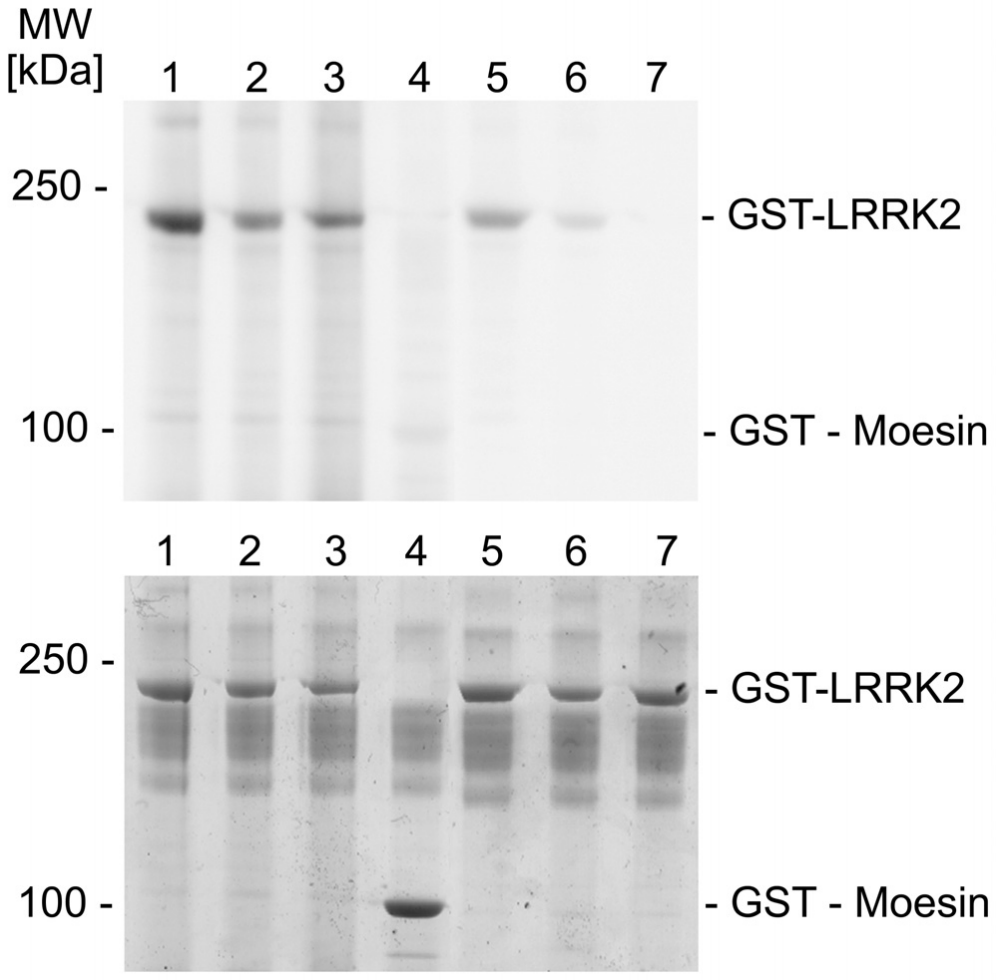
Recombinant protein kinase C zeta phosphorylates a LRRK2 fragment in solution. (Upper panel) Autoradiogram demonstrating phosphorylation of GST-tagged LRRK2(G2019S) (amino acids 970-2527) (lane 1), GST-tagged wildtype LRRK2 (lane 2) and kinase-inactive GST-tagged LRRK2(D1994A) (lane 3) by recombinant protein kinase C zeta *in vitro*. Background autophosphorylation of the three kinases in the absence of protein kinase C zeta is shown in lanes 5–7. GST-tagged moesin is not a substrate for protein kinase C zeta excluding GST phosphorylation by protein kinase C zeta (lane 4). (Lower panel) Coomassie Blue protein staining of the gel shows similar amounts of recombinant LRRK2.

**Figure 5:**
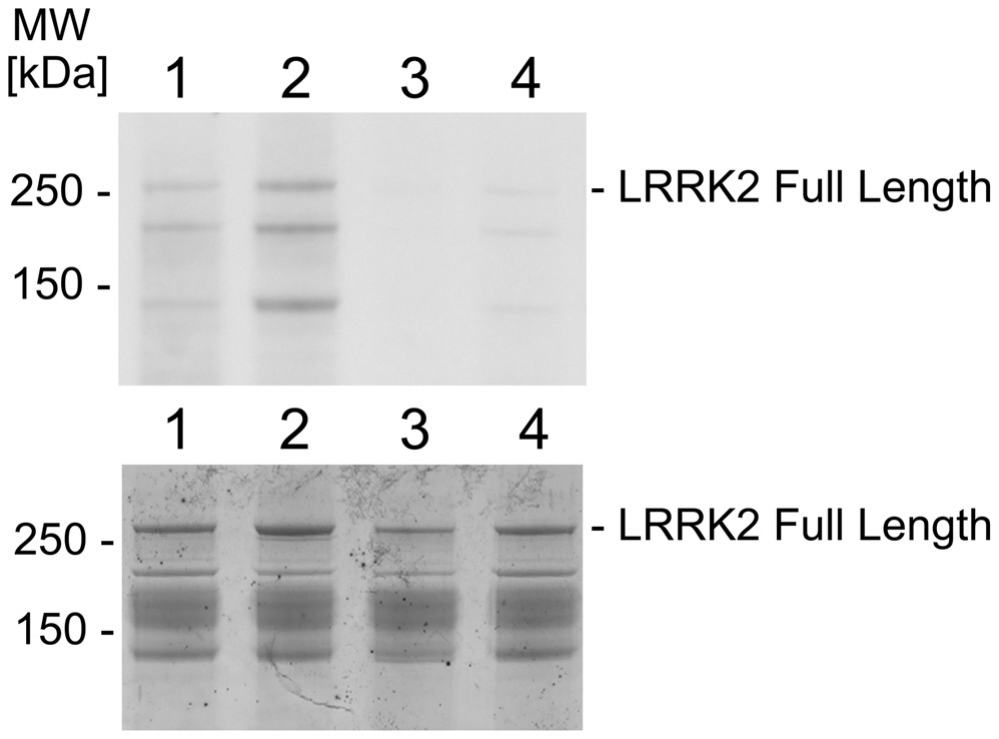
Protein kinase C zeta phosphorylates full-length LRRK2 *in vitro*. (Upper panel) Autoradiogram showing phosphorylation of full-length, wildtype LRRK2 (lane 2, 4) and full-length, kinase-dead LRRK2(K1906M) (lane 1, 3) by recombinant protein kinase C zeta (lane 1, 2) *in vitro*. (Lower panel) Coomassie Blue protein staining shows similar amounts of recombinant LRRK2. Two LRRK2 fragments are visible migrating below 250 kDa that are also phosphorylated by protein kinase C zeta.

**Figure 6:**
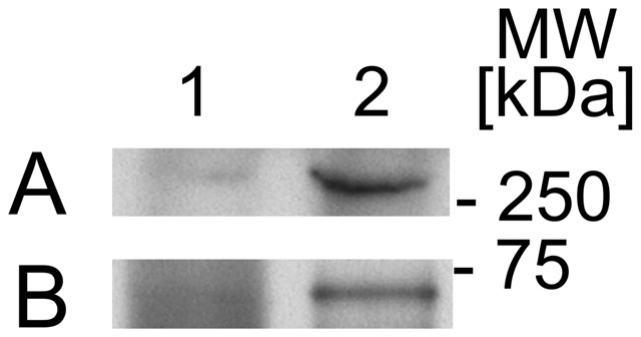
LRRK2 co-immunoprecipitates with protein kinase C zeta from mouse brain. Homogenates of C57BL/6 mouse brains were incubated with an anti-protein kinase C zeta antibody (lane 2) or pre-immune serum as control (lane 1). Immuncomplexes were precipitated using protein G-sepharose beads and subjected to gel electrophoresis and immunoblotting using either an anti-LRRK2 antibody (upper panel) or an anti-protein kinase C zeta antibody (lower panel). Representative results are shown for experiments that were repeated two times.

At this stage of our study, kinase-inactive LRRK2(D1994A) became commercially available. We confirmed that GST-tagged LRRK2(D1994A) lacks autophosphorylation activity in a solution kinase assay (data not shown). Thereafter, substrate identification studies on protein arrays were repeated using active LRRK2(G2019S) and kinase-dead LRRK2(D1994A) as negative control. Surprisingly, protein arrays incubated with inactive LRRK2(D1994A) showed a significant increase in the radioactive signals on the PKC zeta spots compared to buffer controls, which is similar to arrays treated with active kinase (Figure 3C). These findings so far allowed us only to conclude that LRRK2 binds to spotted PKC zeta and becomes phosphorylated by PKC zeta on the array during incubation. This conclusion was tested in a kinase assay in solution. By incubating LRRK2(G2019S), inactive LRRK2(D1994A), or wildtype LRRK2 with recombinant PKC zeta and [γ-^33^P]ATP, we could demonstrate that LRRK2 was efficiently phosphorylated by PKC zeta *in vitro* (Figure 4). In order to exclude that the GST-tag on recombinant LRRK2 is phophorylated by PKC zeta, kinase assays were performed using recombinant GST and GST-tagged proteins leading to negative results (Figure 4). We also confirmed that PKC zeta is able to phosphorylate full-length LRRK2 (Figure 5). To demonstrate *in vivo* interaction between LRRK2 and PKC zeta, we used brain homogenates of wildtype C75BL/6 mice, since PKC zeta is highly expressed in the brain. As shown in Figure 6, endogenous LRRK2 can be co-immunoprecipitated with PKC zeta indicating physiological relevance. The functional consequence of PKC zeta-mediated LRRK2 phosphorylation on LRRK2 enzymatic activity could not be clarified, since PKC zeta efficiently phosphorylated heat-treated moesin (Fig. S1), a validated LRRK2 substrate [14], [15].

**Figure 7:**
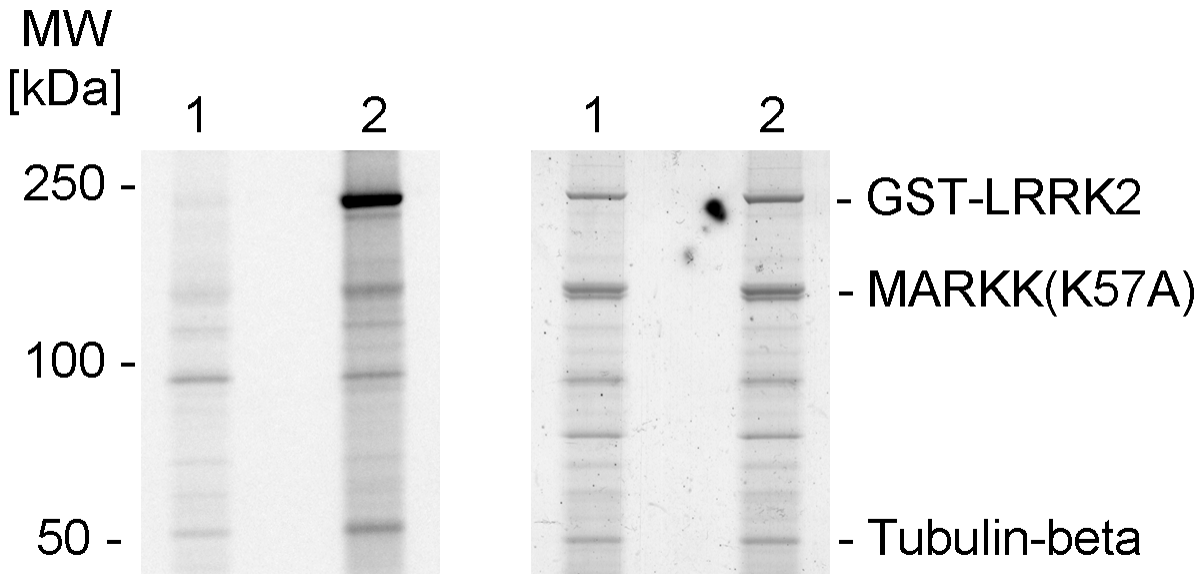
LRRK2 phosphorylates MARKK *in vitro*. (Left panel) Autoradiogram showing phosphorylation of recombinant, kinase-inactive MARKK(K57A) by GST-tagged LRRK2(G2019S) (amino acids 970-2527) (lane 2). Kinase-inactive GST-tagged LRRK2(D1994A) was used as negative control (lane 1). Endogenous tubulin-beta that is co-purified with LRRK2 becomes phosphorylated as well. (Right panel) Coomassie Blue protein staining shows similar amounts of recombinant proteins.

Out of the four arrayed Ste20 serine/threonine kinases that we detected as *in vitro* LRRK2 substrates, TAOK3 is of particular interest. TAO kinases exhibit high sequence homology to microtubule affinity-regulating kinase-activating kinase (MARKK) [16]. MARKK phosphorylates/activates MARK which phosphorylates the microtubule-associated protein Tau [9]. A decrease/increase in Tau phosphorylation has been demonstrated in mouse mutants lacking/overexpressing LRRK2, although Tau is not a direct substrate of LRRK2 [17], [18]. A kinase signaling cascade LRRK2→MARKK→MARK might explain enhanced Tau phosphorylation in brains from LRRK2 overexpressing mice and from human PD patients carrying a gain-of-function LRRK2(G2019S) mutation [2]. To assess phosphorylation of recombinant MARKK by LRRK2 in solution, kinase-dead MARKK(K57A) was incubated with LRRK2(G2019S). As shown in Figure 7, MARKK(K57A) becomes phosphorylated by LRRK2 to a similar extent as tubulin-beta that is co-purified with recombinant LRRK2 [18].

## Discussion

Mutations in LRRK2 represent the most prevalent cause of PD, however the (patho)physiological functions of LRRK2 remain poorly understood. Based on sequence similarities in the conserved kinase domains, LRRK2 has been classified as a member of the receptor-interacting protein (RIP) family kinases that integrate both extracellular and intracellular stress signals [19]. Like RIP1, LRRK2/RIP7 interacts with FADD and TRADD, two central adaptor proteins of death receptors (e.g. TNF-alpha, Fas), indicating that LRRK2 may act within the extrinsic cell death signaling pathway [20]. Imai and colleagues [21] reported that LRRK2 phosphorylates eukaryotic initiation factor 4E-binding protein (4E-BP), a key regulator of protein translation during cell stress, and that LRRK2 genetically interacts with the Rheb GTPase / Tor kinase / 4E-BP signaling pathway in Drosophila. Furthermore, mitogen-activated protein kinase kinases (MKK) 4 and 7 become phosphorylated by LRRK2 *in vitro* suggesting that LRRK2 may represent an upstream component of the MAP kinase signaling cascade [22].

In the present study, we used protein microarrays containing a set of 259 human recombinant proteins that are involved in key signaling networks (including TNF receptor signaling, MAP kinase signaling) in order to identify potential LRRK2 substrates or interactors and to map LRRK2 to specific signaling pathways. Of the published LRRK2 substrates, both 4E-BP and MKK7 are spotted on the arrays, however neither protein became phosphorylated following array incubation with recombinant LRRK2. Recently, Kumar and colleagues [23] reported that the stochiometry of LRRK2-mediated 4E-BP phosphorylation *in vitro* is low and that 4E-BP phosphorylation in HEK293 cells does not change following LRRK2 overexpression suggesting that 4E-BP is not a major substrate of LRRK2. Similarly, we could not detect an increase in phospho-MKK7(S271/T275) levels in LRRK2 overexpressing HEK293 cells by immunoblot analysis (data not shown). Novel potential substrates/interactors of LRRK2 that we identified by protein array analysis included four members of Ste20 serine/threonine kinase family (TAOK3, STK3, STK24, STK25) [24]. Other kinases, like AKT, Raf-1, and PKA have already been shown to bind/phosphorylate STK3 or STK24, thereby modulating kinase activity [25]. Both STK24 and STK25 are highly expressed in the mammalian brain. STK24 kinase activity increases during nerve regeneration and is required for axon outgrowth [26]. In non-neuronal cells, STK24 and STK25 are activated/phosphorylated following oxidative stress promoting apoptotic cell death [27], [28]. It may be hypothesized that the modulation of neurite outgrowth and oxidative stress resistance by LRRK2 shown by others [3], [4], [21] is partly mediated by Ste20 kinases. LRRK2-mediated TAOK/MARKK phosphorylation could be confirmed in an in-solution kinase assay using a kinase-inactive TAOK/MARKK mutant. Thus, the present study may provide the ‘missing link’ of a kinase signaling cascade LRRK2→TAOK/MARKK→MARK→Tau that may partially explain enhanced Tau phosphorylation and axonal pathology in brains from LRRK2 overexpressing mice and human PD patients carrying a gain-of-function LRRK2(G2019S) mutation [4], [7], [9].

LRRK2 has been classified as receptor-interacting protein kinase 7 (RIP7) according to sequence homology in the kinase domain [19]. Our *in vitro* study indicates that LRRK2/RIP7 also shares some functional features with other RIP kinases. Like RIP1-4 [19], LRRK2 interacts with TNF receptor-associated factor 2 which may contribute to LRRK2 ubiquitination and proteasomal degradation in cells. Similar to RIP3 [11], LRRK2 phosphorylates other RIP kinase family members. Finally, we demonstrate that LRRK2 interacts with PKC zeta both on protein arrays and in mouse brain homogenates. While LRRK2 does not efficiently phosphorylate PKC zeta *in vitro*, LRRK2 itself becomes phosphorylated by recombinant PKC zeta. Similar *in vitro* findings have been reported when RIP4 and PKC beta/delta were tested [29]. Consistently, we identified several PKC consensus phosphorylation sites (K/RXXS*/T*) [30] within the LRRK2 protein by sequence analysis. Finally, both PKC zeta (present study) and LRRK2 [14] phosphorylate moesin on threonine-558 suggesting that moesin may act as a common effector for regulation of neurite elongation by brain-specific PKC/PKM zeta and LRRK2, respectively [15], [31].

In summary, our protein microarray analysis functionally links PD-associated LRRK2 to Ste20 kinases and RIP kinases, respectively, and identifies novel LRRK2 interactors/effectors (e.g. STK24, STK25, TAOK/MARKK, PKC zeta) that may contribute to its function in oxidative stress signaling and structural/functional plasticity in neurons.

## Supporting Information

Figure S1 Both LRRK2 and PKC zeta phosphorylate moesin in vitro. (Upper panel) Autoradiogram showing phosphorylation of GST-tagged moesin by co-incubation with: (lane 1 and 2) recombinant PKC zeta and LRRK2(G2019S), (lane 3 and 4) PKC zeta and kinase-dead LRRK2(D1994A), (lane 5 and 6) PKC alone, and (lane 6 and 7) CDK5 as negative control. (Lower panel) Coomassie Blue protein staining shows similar amounts of recombinant GST-tagged moesin.
(0.15 MB TIF)

Table S1 Genepix Pro and ProtoArray Prospector analysis of protein arrays used for kinase substrate identification.
(1.15 MB XLS)

Table S2 Genepix Pro and ProtoArray Prospector analysis of protein arrays used for protein-protein binding studies.
(1.15 MB XLS)
