# Supplementary figures and images for: Signal Transduction Protein Array Analysis Links LRRK2 to Ste20 Kinases and PKC Zeta That Modulate Neuronal Plasticity
Source: PLoS One. 2010 Oct 7;5(10):e13191. doi: 10.1371/journal.pone.0013191 (PMC2951910; doi:10.1371/journal.pone.0013191)

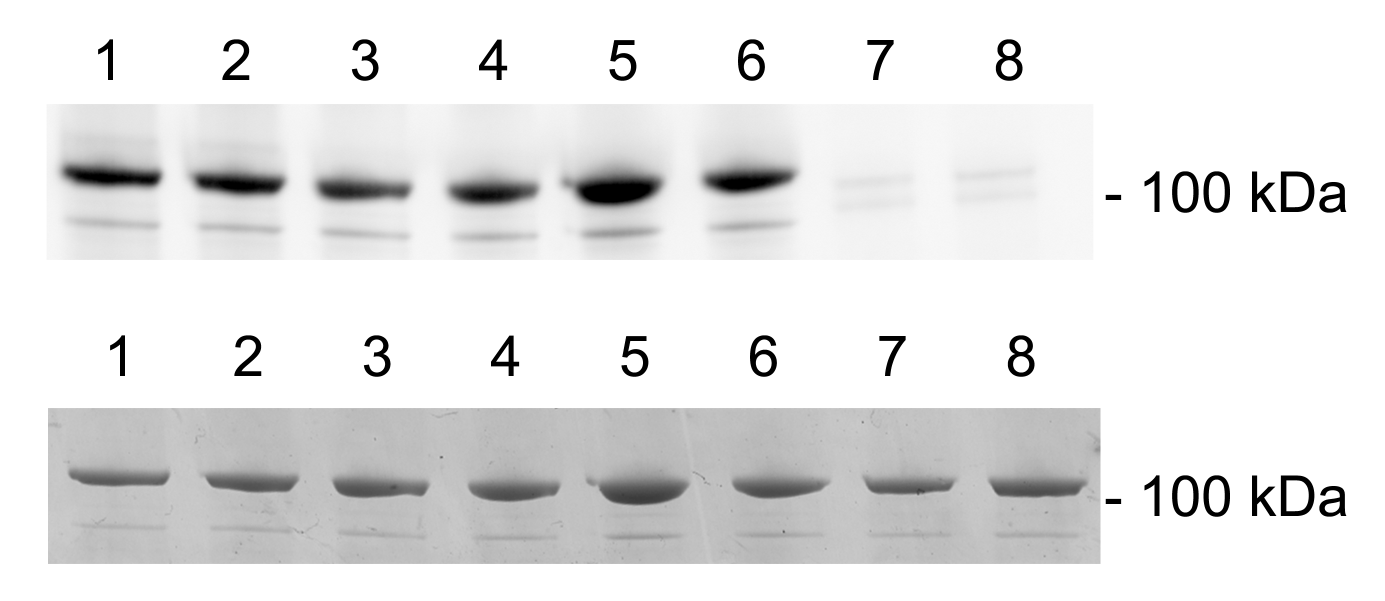

Supplement: Figure S1 — Both LRRK2 and PKC zeta phosphorylate moesin in vitro. (Upper panel) Autoradiogram showing phosphorylation of GST-tagged moesin by co-incubation with: (lane 1 and 2) recombinant PKC zeta and LRRK2(G2019S), (lane 3 and 4) PKC zeta and kinase-dead LRRK2(D1994A), (lane 5 and 6) PKC alone, and (lane 6 and 7) CDK5 as negative control. (Lower panel) Coomassie Blue protein staining shows similar amounts of recombinant GST-tagged moesin. (0.15 MB TIF) [file pone.0013191.s001.tif]
